# Knowledge about Obstetric Danger Signs and Related Factors in Reproductive-Age Women in the Southeast Zone of Tigray, 2021: A Cross-Sectional Study

**DOI:** 10.1155/2022/7346618

**Published:** 2022-06-01

**Authors:** Berhane Teklay Asfaha, Shewit Hailu Gebremariam, Gebremedhin Kinfe Gebremariam, Ataklti Gebretsadik Weldemariam

**Affiliations:** ^1^Department of Midwifery, College of Health Sciences, Assosa University, Assosa, Ethiopia; ^2^Department of Midwifery, College of Medicine and Health Sciences, Adigrat University, Adigrat, Ethiopia; ^3^Department of Pharmacy, College of Medicine and Health Sciences, Adigrat University, Adigrat, Ethiopia

## Abstract

**Background:**

Pregnancy complications are the major health problems among women in developing countries. Globally, around 295,000 women die from pregnancy-related causes annually and 86% of these maternal deaths happen in developing countries.

**Objective:**

To assess knowledge of obstetric danger signs among reproductive-age women living in southeastern zone of Tigray Region, Ethiopia, 2021.

**Methods:**

A community-based quantitative cross-sectional survey was undertaken in southeastern zone of Tigray. A multistage random sampling technique was implemented to select total participants of 410 reproductive-age women. Two districts were randomly selected, and from those districts, 12 kebeles were selected randomly, and the calculated sample size (410) was proportionally allocated to each selected kebel. The data were collected by using face-to-face interview with a structured questionnaire from January 20 to February 20/2021 after ensuring that all requirements of ethical considerations were fulfilled. The collected data were entered into EpiData version 4.2 and then exported to SPSS version 20 for analysis. Descriptive statistics with frequency, percentage, table and graph, and cross-tabulation were used for presentation of result. Bivariable and multivariable analyses were used to examine the association. Odds ratios with 95% confidence interval and *P* value < 0.05 were used to determine the statistical association.

**Result:**

Four hundred ten reproductive-age women participated in the study making a response rate of 100%. Leakage of fluid per vagina was the most commonly mentioned obstetric danger signs (61%). Overall, one hundred seventy-two (42%) had good knowledge on obstetric danger sign. Educational status of the mother (AOR (95%CI = 2.7 (1.189-6.24))), site of delivery (AOR (95%CI = 2.2 (1.6-3.432))), and having history of an ANC follow-up (AOR (95%CI = 2.4 (1.13-5.6))) were found to be independent predictors of knowledge of women about the obstetric danger sign. *Conclusion and Recommendation*. Educational status of the mother site of delivery and having history of an ANC follow-up were independently associated with knowledge of women about obstetric danger signs. Thus, provision of the Information, Education and Communication targeting women, family, and the general community on obstetric danger signs and associated factors was recommended.

## 1. Introduction

Pregnancy-related complications are the common health problems among women in developing countries. Globally, around 295,000 women die from pregnancy-related causes annually and 86% of these maternal deaths happen in developing countries [1].

The classification of countries into the developed and developing countries is based on economic status such as GDP, GNP, income per capita, industrialization, and standard of living. Developed countries refer to the sovereign state whose economy is highly advanced and possesses large technological infrastructure compared to other nations, while the countries with low industrialization and low human development index are called developing countries [2].Therefore, maternal mortality rates are highest in developing countries, mainly due to deficiencies in the social, economic, and political conditions of the countries involved, combined with a grossly inadequate quantity and quality of available health services [3].

Although there was a significant progress in all developing regions, the average annual percentage decline in the global maternal mortality ratio (MMR) was 2.9%; this means that, on average, the global MMR declined by 2.9% every year between 2000 and 2017 [4].

Every year, about 500,000 women worldwide die due to complications associated with pregnancy, and unfortunately, maternal aspects of maternal child health have all too often been relegated to secondary priority within the child surviva1 revolution [5].

Developing countries account for 99% (284,000) of the global maternal deaths, the majority of which are in sub-Saharan Africa (162,000) and Southern Asia (83,000). These two regions accounted for 85% of global burden, with sub-Saharan Africa alone accounting for 56%.The average maternal mortality ratio in developing countries in 2010 was 240 per 100,000 births versus 16 per 100,000 in developed countries reflecting inequities in access to health services and highlighting the gap between rich and poor. Sub-Saharan Africa had the highest maternal mortality ratio at 500 maternal deaths per 100,000 live births [6]. According to a systematic analysis of progress towards Millennium Development Goal 5, more than 50% of all maternal deaths in 2008 were in only six countries (India, Nigeria, Pakistan, Afghanistan, Ethiopia, and the Democratic Republic of the Congo) [4].

The situation in Ethiopia is similar to the situation in many developing countries. In Ethiopia, the levels of maternal mortality and morbidity are among the highest in the world and the current estimate of MMR is 412 per 100,000 live births. That is, for every 1,000 births in Ethiopia, there are about 4 maternal deaths [7].

Reductions of mortality and morbidity of both the mother and newborn have been identified as priority areas that need urgent attention by the health sector. Maternal morbidity and mortality could be prevented and minimized significantly if women and their families recognize obstetric danger signs and promptly seek health care [8].

The commonest danger signs during pregnancy include severe vaginal bleeding, swollen hands/face, and blurred vision. Key danger signs during labor and childbirth include severe vaginal bleeding, prolonged labor, convulsions, and retained placenta. Danger signs during the postpartum period include severe bleeding following childbirth, loss of consciousness after childbirth, and fever. Hemorrhage remains the leading cause of maternal mortality, accounting for approximately one-third of deaths. Many of the complications that result in maternal deaths contributing to prenatal deaths are unpredictable, and their onset can be both sudden and severe. The complications leading to maternal death can occur without warning at any time during pregnancy and childbirth. Low awareness of danger signs and symptoms during pregnancy, labor, delivery, and postpartum contributes to delays in seeking and receiving skilled care. Awareness of the danger signs of obstetric complications is the essential first step in accepting appropriate and timely referral to obstetric and newborn care [9].

Knowledge of obstetric danger signs and birth preparedness are strategies aimed at improving the utilization of skilled care during low-risk births and emergency obstetric care in complicated cases in low-income countries. Increased knowledge and awareness is essential for reducing delays in seeking health care and in reaching a health facility [10].

Communities and women should be empowered not only to recognize pregnancy-related risks but they must also have the means to react quickly and effectively once such problems arise [11]. The national reproductive strategy of Ethiopia has given emphasis to maternal and newborn health so as to reduce the high maternal and neonatal mortality. The strategy focuses on the need to empower women, men, families, and communities to recognize pregnancy-related risks and to take responsibility for developing and implementing appropriate response to them. One of the targets in the strategies is to ensure that 80% of all families recognize at least three danger signs associated with pregnancy-related complications by 2010 in areas where health extension program is fully implemented [12].

Maternal mortality is the leading cause of the adult female deaths in many countries. Women's death during childbirth often means death for the newborn, and both death and disabilities translate into emotional, social, and economic hardships for women's older children, their entire families, and even for communities [13].

Every day, 810 women die from preventable causes related to pregnancy and childbirth [14]. Maternal deaths are avoidable, skilled care before, during and after child birth, and if women with complications are able to identify and seek appropriate emergency obstetric care which makes a difference between life and death [10]. Maternal deaths have both direct and indirect causes. Around 73% of maternal deaths worldwide are brought about by direct obstetric complications.

The five major global causes of maternal death are as follows: severe bleeding (mostly bleeding postpartum), infections (also mostly soon after delivery), unsafe induced abortion, hypertensive disorders in pregnancy (eclampsia), and obstructed labor. Globally, about 73% of maternal deaths are due to these causes. Hemorrhage alone accounts for one-third of all maternal deaths in Africa, yet many of these deaths are preventable. Severe bleeding after birth can kill a healthy woman within two hours if she is unattended. Obstetric fistula resulting from obstructed labor is a long-term complication suffered by as many as two million women. Indirect causes such as malaria, diabetes, hepatitis, anemia, and other cardiovascular disorders which are aggravated by pregnancy can also lead to maternal death [15].

Knowledge of the danger signs of obstetric complications is the important first step in accepting applicable and timely referral to obstetric and newborn care. Increasing awareness of women on danger signs of pregnancy, childbirth, and the postpartum period improves mother's attitude to seek medical care and is key for safe motherhood [16].

When mothers do not recognize the danger signs in pregnancy, adverse effects can occur to the mother, the unborn baby, or the pregnancy itself. Adverse effects include the following: illness or death of the mother; for instance, severe bleeding can lead to anemia or death of the mother and infection to the unborn baby through prematurely ruptured membranes, when amniotic fluid leaks from the vagina. If not attended to, this can lead to fetal or neonatal morbidity and mortality, termination of a pregnancy before term in vaginal bleeding. Maternal hypertension or 4 fevers can lead to increased numbers of neonatal deaths or prematurely born babies who may eventually die due to inadequate facilities to care for them [17].

A mother's death in childbirth denies her children their natural, primary care giver and significantly increases the risk that her infant will die or fail to survive to age 5. A mother's death also has an extremely detrimental effect on her children's access to education and health care. Many children who survive without mothers also risk being emotionally lost [18]. Most maternal deaths are avoidable, as the health care solutions to prevent or manage complications are well known. All women need access to antenatal care in pregnancy, skilled care during childbirth, and care and support in the weeks after childbirth. It is particularly important that all births are attended by skilled health professionals, as timely management and treatment can make the difference between life and death [18, 19].

According to the Ethiopian demographic health survey 2016, only 26% of the deliveries is attended in health facility. In Ethiopia, the maternal mortality rate is 412 per 100,000 live, IMR 48/1000 and NMR 29/1000 live births, the highest in the world. In Ethiopia, there is little information about the Knowledge, Attitude and Practice of obstetric danger signs during pregnancy since the introduction of health extension workers (HEWs), despite the national reproductive strategy aims to raise the awareness to 80% in the area in which HEW are deployed [7].

A study conducted in Tsegedie District indicated that the knowledge level of women about obstetric danger signs was low and affected by residential area. Therefore, the identified deficiencies in awareness should be addressed through maternal and child health services by designing an appropriate strategies including provision of targeted Information, Education and Communication. In spite of great potential of the Knowledge, Attitude and Practice of obstetric danger signs in reducing the maternal and newborn deaths its status are not well known in most of sub-Saharan Africa including Ethiopia [20]. The aim of this study is therefore to close this gap by raising the current state of knowledge on danger signs among mothers in the study area.

## 2. Methods

### 2.1. Study Setting

Tigray Regional State is one of the nine regional states of Ethiopia, which is found in the northern part of Ethiopia. The capital city of Tigray is Mekelle City which is 781 km far from Addis Ababa.

The study area was in the southeastern zone of Tigray Regional State. It is bordered on the south by southern zone, on the southeast by Amhara Region, on the northeast by central zone, on the north by eastern zone, and on the east by the Afar region. The latitude and longitude of the administrative centers of the districts are as follows: Enderta (Quiha) with latitude and longitude of 13°28′37^″^ N, 39°32′42^″^ E with an elevation of 2247 meters above sea level; Deguatemben (H/selam) with latitude and longitude of 13°39′ N, 39°10′ E with an elevation of 2650 meters above sea level; H/Wajirat (Adi Gudem) with latitude and longitude of 13°15′ N, 39°31′ E with an elevation of 2100 meters above sea level; and Samre with latitude and longitude of 13°11′ N, 39°12̍′ E with elevation of 1855 meters above sea level.

The estimated size of population according to 2007 census conducted by the CSA is 392,142, of which 21,125 or 5.39% are urban dwellers [21]. The zone has four woredas (Saharti Samre, Hintalo Wajirat, D/temben, and Enderta) with a total of 87 kebeles, 23 of which are found in Saharti Samre, 23 in Hintalo Wajirat, 24 in Deguatemben, and 17 in Enderta. It also contains seven primary hospitals, 25 health centers, and 83 health posts.

The study will be conducted from January 20 to February20/2021 in the southeastern zone of Tigray.

### 2.2. Participants

The sampled reproductive-age women living in the southeastern zone of Tigray and who fulfill the inclusion criteria were taken as the study population. All reproductive-age-group women who were mentally stable, those who were available at the time of data collection period, and women who were not severely ill were included, while reproductive-age women who are health professionals or working on health and health-related fields were excluded.

### 2.3. Sample Size Determination and Sampling Technique

The sample size was calculated using a single population proportion formula with the following assumption: prevalence of awareness on risk factor for obstetric fistula was 50%, 95% of confidence interval (1.96), 5% margin of sampling error tolerated, 5% of nonresponse rate, and then the final sample size was 410. Random sampling technique was used to select the total of 410 study participants.

In the southeastern zone of Tigray, there are four woredas, namely, Enderta woreda, Saharti Samre, Hintalo Wajirat, and Deguatemben. From those four woredas, two woredas were selected using a simple random sampling. Accordingly, Hintalo Wajirat and Enderta were selected. There are 23 kebeles in Hintalo Wajirat and 17 kebeles in Enderta woreda with a total of 39 kebeles. Six kebeles were selected from 23 in Hintalo Wajirat and six kebeles from 17 kebeles in Enderta by using a simple random sampling.

Maernet, Dedba, Arato, and Meseret were selected from Enderta, and Mesanu, Hiwane, M/Nebri, and Hareko were included from H/Wajirat.

The calculated sample size (410) was proportionally allocated to each selected kebeles in both woredas.

A list of reproductive-age women was obtained from HEWs. Then, individual units were selected by using a simple random sampling technique to obtain the required data.

### 2.4. Study Variables

#### 2.4.1. Dependent Variable

- Knowledge on obstetric danger sign

#### 2.4.2. Independent Variables

- Sociodemographic factors (age, marital status, religion, women education, occupation, age of marriage, and residence)
- Obstetric factors
- Other factors (source of information and distance to health institution)

#### 2.4.3. Definition of Terms

- *Danger signs*: refers to presence of condition that increases the chances of pregnant woman and/or her unborn child dying or having poor health
- *Knowledge*: refers to knowledge of obstetric danger signs means the basic information that the mothers have regard obstetric danger signs
- *Good knowledge*: refers to those participants who respond correctly to knowledge questions and score above the mean value
- *Poor knowledge*: refers to those participants who correctly respond to knowledge questions and score below the mean value

### 2.5. Data Collection Tools and Techniques

For data collection, a structured face-to-face questionnaire English version will be adapted after a review of different literatures and modified depending on the local situation and the research objective. Initially, it was developed in English and it was translated to Amharic by an individual who is native to Amharic language. And it was translated back to English language by another individual in order to maintain its consistency. The questionnaire contained questions on sociodemographic characteristics, source of health-related information, history of contraceptive use, obstetric history, and questions on knowledge on obstetric danger sign. Five midwives with diploma and three BSc level health professionals were recruited and trained as data collectors and supervisors, respectively. Eligible participants were approached and requested to consent voluntarily to participate into the study. Upon consenting, a study number with a code was assigned for identification. Inclusion into the study was done by using proportion from the kebeles and then randomly select until the required sample size will be achieved using a face-to-face structured questionnaire.

### 2.6. Data Quality Assurance and Control

The data collection instrument was pretested for accuracy of responses, language transparency, correctness of data collection tools, and estimation of the time required, and the necessary amendment was considered based on it prior to the actual data collection. It was carried out one week proceeding to the actual data collection period, in five percent of the nonstudy participants that fulfill the inclusion criteria. In addition, the data collectors were trained for one day on the techniques of data collection. The training also included importance of disclosing the possible benefit and purpose of the study to the study participants before the start of data collection. Maintaining confidentiality of the participants throughout the whole process of data collection was discussed and ascertained during the training. The researcher was checked for completeness and consistency of questionnaires filled by the data collectors to ensure the quality of the data and also visit the data collectors as many times as possible to check whether he/she collects the data appropriately. The researcher was also appraising the data during the data analysis stage to verify the completeness of the collected data.

### 2.7. Data Processing and Analysis

After data collection, EpiData version 4.2 was used for entry and then exported to SPSS version 20 for analysis. Binary logistic regression model was used to determine the factors associated with obstetric fistula. Those variables which were clinically important and having a *P* < 0.25 in bivariate analysis were the candidate variables for the final multivariable binary logistic regression model. The crude and adjusted measures of effect odds ratio were determined with 95% CI, and *P* < 0.05 was used to declare statistical significance. Goodness of fit of the final model was assessed using the Hosmer and Lemeshow goodness of fit test, and multicolinearity was checked. Finally, the result was presented using tables, figures, and texts.

## 3. Results

### 3.1. Sociodemographic Character of the Study Participants

The response rate of the study participants was 100%. The mean age and standard deviation of the study participants were 36.51 and ±9.712 (ranging from 15 to 49) years, respectively. Most of the study participants (37.1%) were found in the age of ≥45 years, and with regard to religion, most of them (75.1%) (308/410) were orthodox followers. Regarding educational status, about 57.1% (234/410) of the participants were illiterates. About marital status, the majority (69.8%) of the participants were married (Table 1).

### 3.2. Past Obstetric Characteristics of Respondents

Most of the study participants (67.8%) had history of pregnancy, and regarding the frequency of pregnancy, most of them (52.9%) (148/410) had above four times. Regarding the history of intrauterine fetal death, the majority (92.1%) (258/280) of the participants had not. About time taken to reach health institution, the majority (75.6%) (310/410) of the participants take ≥30 minutes to reach health facility. About source of information, 76.8% have been gotten information about obstetric danger sign from health workers (Table 2).

### 3.3. Knowledge about Obstetric Danger Signs

Knowledge of respondents about delivery services was assessed by questions of danger signs related to pregnancy and childbirth. When the participants were asked to mention obstetric danger sign, the most common spontaneously mentioned danger signs were leakage of fluid per vagina by 242 (59%), body swelling by 244 (59.5%), absence or decrease of FHB by 187 (45.6%), prolonged labor by 234 (57%), excessive vaginal bleeding by 245 (60%), and retained placenta by 166 (40.5%). Overall, one hundred seventy-two (42%) of respondents mentioned above mean value of obstetric danger signs, and two hundred thirty-eight (58%) mentioned below mean value regarding obstetric danger sign (Table 3).

### 3.4. Associated Factors of Knowledge on Obstetric Danger Sign

Binary logistic regression was done to identify significant factors with the knowledge on obstetric danger sign than those factors with a *P* value of less than 0.25 during bivariate analysis had been taken to multivariable analysis. The variables significantly associated in bivariate analysis were educational level, marital status, history of an ANC follow-up, age during first pregnancy, total number of birth, total live birth, site of delivery, and frequency of pregnancy. The multivariable binary logistic regression analysis showed the following finding. Educational level, site of delivery, and history of an ANC follow-up were independently associated with knowledge on obstetric danger sign. Reproductive-age women who had a diploma and above were 2.7 times more likely to have good knowledge on obstetric danger sign as compared to those reproductive-age women who were illiterate (AOR (95%CI = 2.7 (1.189-6.24))).Women who have history of an ANC follow-up were 2.4 times more likely to have good knowledge on obstetric danger sign as compared to their counterparts (AOR (95%CI = 2.4 (1.13-5.6))). Moreover, reproductive-age women who had history of site of delivery were 2.2 times more likely to have good knowledge on obstetric danger sign as compared to those who had not (AOR (95%CI = 2.4 (1.3-5.3))) (Table 4).

## 4. Discussion

This community-based cross-sectional study identified factors that influence knowledge about obstetric danger signs among reproductive-age women in southeastern zone of Tigray.

According to this study, around 42% (37-48.4%) of participants had good knowledge about obstetric danger sign which was higher than the study conducted in Tanzania and Zambia [22, 23] which were 25.2% and 29%, respectively. The difference could be that due to difference in study year, there is expected to increase knowledge on obstetric danger sign from time to time due to improvement in access and utilization of the health care information being provided. But it was lower as compared to studies conducted in Madagascar and Egypt [24, 25] which were 51.9% and 79.6%, respectively. This difference might be due to difference in study setting; this study was community based, but the previous studies were institution based which they can access health information given by health professionals. This study shows there is a significant association between women's knowledge on obstetric danger sign and their level of education; women who have diploma and above level of education were 2.7 times (AOR (95%CI = 2.7 (1.189-6.24))) more likely to have good knowledge on obstetric danger sign as compared to those reproductive-age women who were not educated/illiterate. This is due to the fact that educated women were more likely to utilize an institutional delivery service compared with uneducated women; this may be partly better informed about obstetric complications and made them well informed regarding obstetric danger sign. This finding was supported by the study conducted in Tsegedie District, Western Tigray Region [20]. Those who had delivered at health institution were 2.2 times (AOR (95%CI = 2.2 (1.3-5.3))) more likely to have good knowledge on obstetric danger sign as compared to those who had history of home delivery. This can be explained by the fact that increased awareness among women who had history of institutional delivery may be related to the health-related education given by health care professionals. The other strong predictor of knowledge of women about the obstetric danger signs was a place history of ANC follow-ups. The likelihood of being knowledgeable was 2.4 times (95%CI = 2.4 (1.13-5.6)), higher when the mothers had history of an ANC follow-up. This can be explained by the fact that increased awareness among women who had history of an ANC follow-up increased knowledge among women who had history of an ANC follow-up may be related to the health-related information and education given by health care professionals during each visit especially those who had complications associated with their pregnancy. And it is consistent with the study conducted in Madagascar [24].

Key obstetric danger signs assessed included were leakage of fluid per vagina, body swelling, absence or decrease of FHB, prolonged labor, excessive postpartum vaginal bleeding, and retained placenta. In line with the findings from Tanzania, Madagascar, and Tsegedie District [24, 26, 27], this study indicated that greater than half (60%) of the study subjects mentioned vaginal bleeding as a danger sign. This study was supported by a study conducted in Tanzania [26].

## 5. Conclusions and Recommendation

The study result suggests that the majority of reproductive-age women in the study area had poor knowledge regarding obstetric danger sign. Moreover, educational status of the mother, site of delivery, and having history of an ANC follow-up were independently associated with knowledge of women about obstetric danger signs. Thus, provision of the Information, Education and Communication targeting women, family, and the general community on obstetric danger signs and associated factors was recommended.

## Figures and Tables

**Table 1:** Sociodemographic characteristics of respondents among reproductive-age women in southeastern zone of Tigray, Ethiopia, 2021 (*N* = 410).

Variable	Category	Frequency	Percentage (%)
Age	15-24	82	20
	25-34	92	22.4
	35-44	84	20.5
	≥45	152	37.1
	Total	410	100

Religion	Orthodox	308	75.1
	Muslim	102	24.9
	Total	410	100

Marital status	Single	108	26.3
	Married	286	69.8
	Widowed	16	3.9
	Total	410	100

Ethnicity	Tigraweyty	378	92.2
	Afar	32	7.8
	Total	410	100

Educational status	Illiterate	234	57.1
	Read and write	42	10.2
	Elementary school	32	7.8
	Secondary school	44	10.7
	Diploma and above	58	14.1
	Total	410	100

Occupation	Housewife	254	62
	Government employed	72	17.6
	Student	66	16.1
	Merchant	18	4.4
	Total	410	100

Income	≤500	246	60
	501-1000	76	18.5
	1001-1500	24	5.9
	1501-2000	30	7.3
	≥2001	34	8.3
	Total	410	100

Husband occupation (*N* = 310)	Farmer	144	46.45
	Government employed	44	14.19
	Private employed	58	18.7
	Merchant	64	20.65
	Total	310	100

Residence	Rural	340	83
	Urban	70	17
	Total	410	100

**Table 2:** Past obstetric characteristics of mothers aged 15–49 years in southeastern zone, Tigray Region, Northwest Ethiopia, 2021.

Variable	Category	Frequency	Percentage (%)
Ever been pregnant	Yes	278	67.8
	No	132	32.2

Frequency of pregnancy (*N* = 280)	One time	110	39.3
	2-4 times	22	7.9
	Above 4 times	148	52.9
	Total	280	100

Age during first pregnancy (*N* = 280)	<22	138	49.3
	22-29	30	10.7
	≥30	112	40
	Total	280	100

Hx of an ANC follow-up	Yes	222	80
	No	56	20
	Total	278	100

Site of delivery	Health institution	216	78
	Home	52	32

Total no. of birth (*N* = 280)	One	32	11.4
	Two	114	40.7
	Above two	134	47.9
	Total	280	100

History of IUFD (*N* = 280)	Yes	22	7.9
	No	258	92.1
	Total	280	100

Time taken to reach heath institution	<30 minutes	100	24.4
	≥30 minutes	310	75.6

Source of information about obstetric danger sign	Health worker	315	76.8
	Media	95	23.2

**Table 3:** Knowledge of the obstetric danger signs among mothers aged 15–49 years in southeastern, Tigray Region, North Ethiopia, June 2021 (*n* = 410).

Variable	Category	Frequency	Percentage
Prenatal vaginal bleeding	Yes	245	60
	No	165	40
	Total	410	100

Leakage of fluid	Yes	242	59
	No	168	41
	Total	410	100

Body swelling	Yes	244	59.5
	No	166	40.5
	Total	410	100

Persistent headache and visual disturbance	Yes	207	50.5
	No	203	49.5
	Total	410	100
Prolonged labor	Yes	234	57
	No	176	43

Absence or decrease of FHB	Yes	187	45.6
	No	223	54.4
	Total	410	100

Retained placenta	Yes	166	40.5
	No	244	59.5
	Total	410	100

Excessive postpartum vaginal bleeding	Yes	245	60
	No	165	40
	Total	410	100

**Table 4:** Associated factors of knowledge on obstetric danger sign among reproductive-age women in southeastern zone of Tigray, Ethiopia, 2021.

Variable	Category	Knowledge		COR (95% CI)	AOR (95% CI)
		Good	Poor		
Educational level	Illiterate	76	158	[1]	[1]
	Read and write	16	26	1.27 (0.69-3.12)	1.2 (0.53-3.23)
	Primary education	3	29	0.23 (0.12-1.5)	0.86 (0.295-1.334)
	Secondary education	23	21	7.8 (3.028-13.49)^∗∗^	2.2 (0.877-5.48)
	Diploma and above	50	8	13 (9.475-19.119)^∗∗^	**2.7** (**1.189**-**6.24**)^∗^

Marital status	Married	133	153	1.82 (1.22-4.3)^∗^	2.2 (0.342-3.432)
	Single	35	73	[1]	[1]

Frequency of pregnancy	One	41	69	[1]	[1]
	Two-four	12	10	2 (1.08-5.76)^∗^	1.23 (0.87-4.32)
	Above four	81	69	1.98 (1.12-3.35)^∗^	3.3 (1.12-4.8)^∗∗^

History of an ANC follow-up	Yes	120	102	2.12 (1.3-4.8)^∗∗^	**2.4** (**1.13**-**5.6**)^∗∗^
	No	20	36	[1]	[1]

Age during first pregnancy	<22 years	41	97	[1]	[1]
	22-29 years	20	10	4.73 (0.56-7.2)	2 (0.89-5.543)
	≥30 years	73	39	4.43 (0.793-7.98)	3.21 (0.896-8.43)

Frequency of birth	1-4	39	69	8.6 (5.57-13.293)^∗∗^	1.6 (0.761-3.201)
	>4	95	77	[1]	[1]

Total live birth	1	14	18	[1]	[1]
	2	35	79	0.57 (0.12-2.42)	0.3 (0.1-5)
	>2	85	49	2.25 (1.32-7.7)^∗^	1.5 (0.13-6.2)

Site of delivery	Institution	216	78	1.7 (1.02-5.23)^∗^	**2.2** (**1.3**-**5**.3)^∗∗^
	Home	52	32	[1]	[1]

^∗^Significant at *P* value < 0.05. ^∗∗^Significant at *P* < 0.001. [1]: reference.

## Data Availability

The datasets used during the current study are available from the corresponding author on reasonable request.

## Abbreviations

ANC: Antenatal care
AOR: Adjusted odds ratio
CI: Confidence interval
COR: Crude odds ratio
CSA: Central Statistical Agency
DHS: Demographic and Health Survey
EDHS: Ethiopian Demographic and Health Survey
HEW: Health extension worker
IEC: Information, Education and Communication
IMR: Infant mortality rate
JHPIEGO: Johns Hopkins Program for International Education in Gynecology and Obstetrics
KAP: Knowledge, Attitude and Practice
MDG: Millennium Development Goal
MMR: Maternal mortality rate
MOH: Ministry of Health
NMR: Neonatal mortality rate
SPSS: Statistical Package for the Social Sciences
TBA: Traditional birth attendant
TV: Television
UNICEF: United Nations Children's Fund
WHO: World Health Organization
ETB: Ethiopian birr
IUFD: Intrauterine fetal death.
